# Inflammation and resolution signaling in cardiac repair and heart failure

**DOI:** 10.1016/j.ebiom.2022.103992

**Published:** 2022-04-08

**Authors:** Ganesh V. Halade, Dae Hyun Lee

**Affiliations:** Division of Cardiovascular Sciences, Department of Medicine, Heart Institute, University of South Florida, 560 Channelside Dr, Tampa, FL 33602, United States

**Keywords:** Cardiac remodeling, Cyclooxygenase, Efferocytosis, Heart failure, Inflammation, Leukocytes, Lipoxygenase, Non-resolving inflammation, Polyunsaturated fatty acids, Resolution of inflammation, Specialized pro-resolving mediators

## Abstract

Unresolved inflammation is a key mediator of advanced heart failure. Especially, damage, pathogen, and lifestyle-associated molecular patterns are the major factors in initiating baseline inflammatory diseases, particularly in cardiac pathology. After a significant cardiac injury like a heart attack, splenic and circulating leukocytes begin a highly optimized sequence of immune cell recruitment (neutrophils and monocytes) to coordinate effective tissue repair. An injured cardiac tissue repair and homeostasis are dependent on clearance of cellular debris where the recruited leukocytes transition from a pro-inflammatory to a reparative program through resolution process. After a cardiac injury, macrophages play a decisive role in cardiac repair through the biosynthesis of endogenous lipid mediators that ensure a timely tissue repair while avoiding chronic inflammation and impaired cardiac repair. However, dysregulation of resolution of inflammation processes due to cardiometabolic defects (obesity, hypertension, and diabetes), aging, or co-medication(s) lead to impaired cardiac repair. Hence, the presented review demonstrates the fundamental role of leukocytes, in particular macrophages orchestrate the inflammation and resolution biology, focusing on the biosynthesis of specialized lipid mediators in cardiac repair and heart failure. This work was supported by research funds from National Institutes of Health (AT006704, HL132989, and HL144788) to G.V.H. The authors acknowledges the use of Servier Medical Art image bank and Biorender that is used to create schematic Figures 1–3.

## Introduction

**Figure 1:**
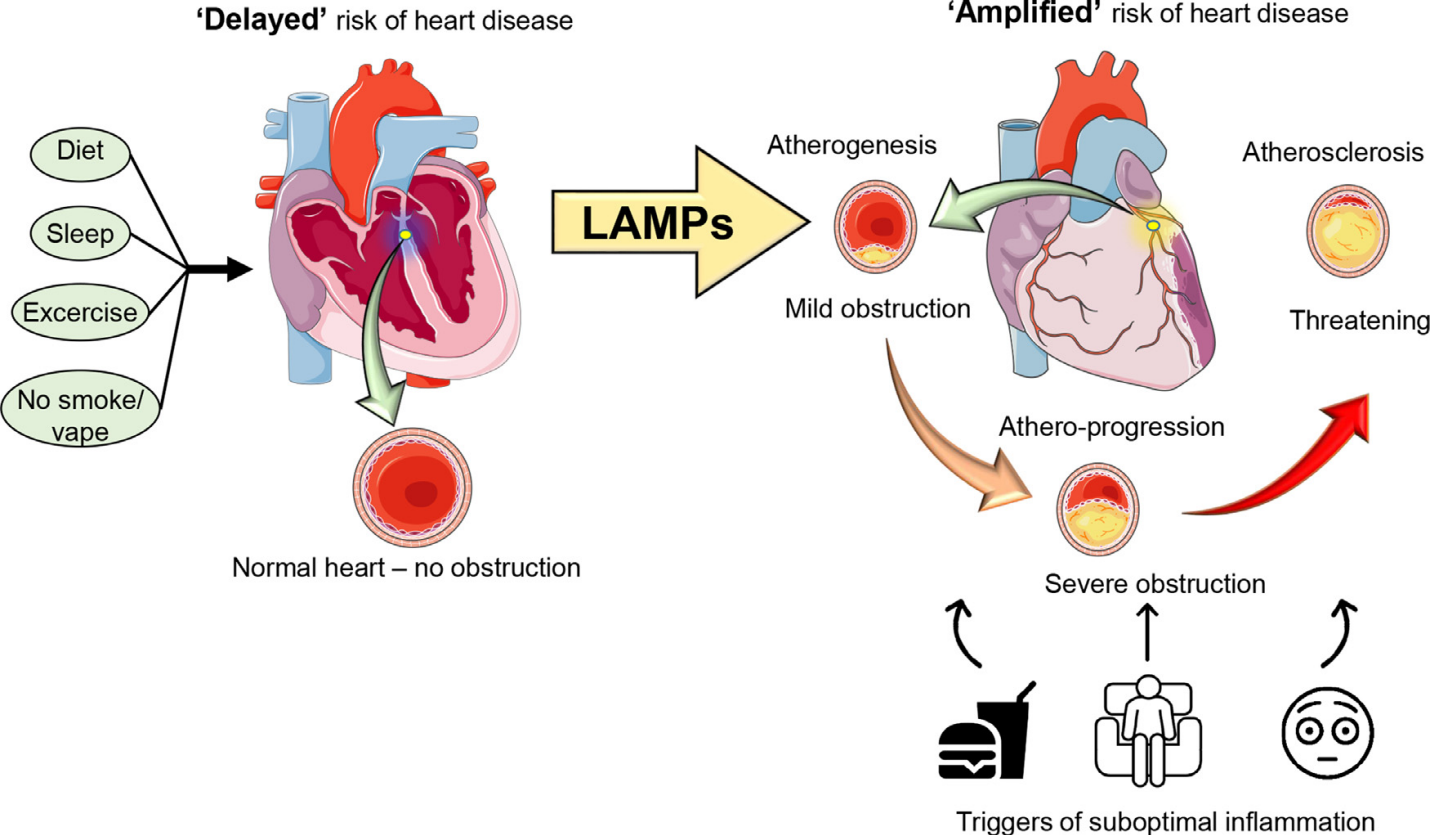
**Progression of a healthy heart to a diseased heart.** Lifestyle-associated molecular patterns (LAMPs) begins with the initial build of atheroma in a coronary artery that progresses to advanced atherosclerosis if not managed on time. Ideal adherence to healthy diet, sleep, and physical activity (exercise) ‘delays’ the risk of heart disease, in contrast, unhealthy diet, sedentary lifestyle, inadequate sleep with mental stress leads to lifestyle-associated molecular patterns that ‘amplify’ and perpetuate suboptimal inflammation. Lifestyle-associated risk factors influence plaque composition (atherogenesis to atheroprogression), leukocyte activation, and immune cell population by modulating traditional and novel pathways in cardiovascular disease.

According to the American College of Cardiology/ American Heart Association, the most important way to prevent cardiovascular disease, including coronary artery disease and heart failure (HF) is to promote a healthy lifestyle which includes healthy diet, sufficient exercise, good sleep pattern.^1^ In contrast, dysregulation of diet, sleep, and exercise aggravate variety of molecular patterns in heart and other immune responsive organ (Figure 1).^2^, ^3^, ^4^ Recently, suboptimal or residual inflammation is gaining the attention as a responsible factor in the progression of cardiovascular disease (CVD). Thus, it is important to revisit the importance of inflammation in the genesis of cardiovascular disease and consider new strategies for prevention and treatment.^5^

**Figure 2:**
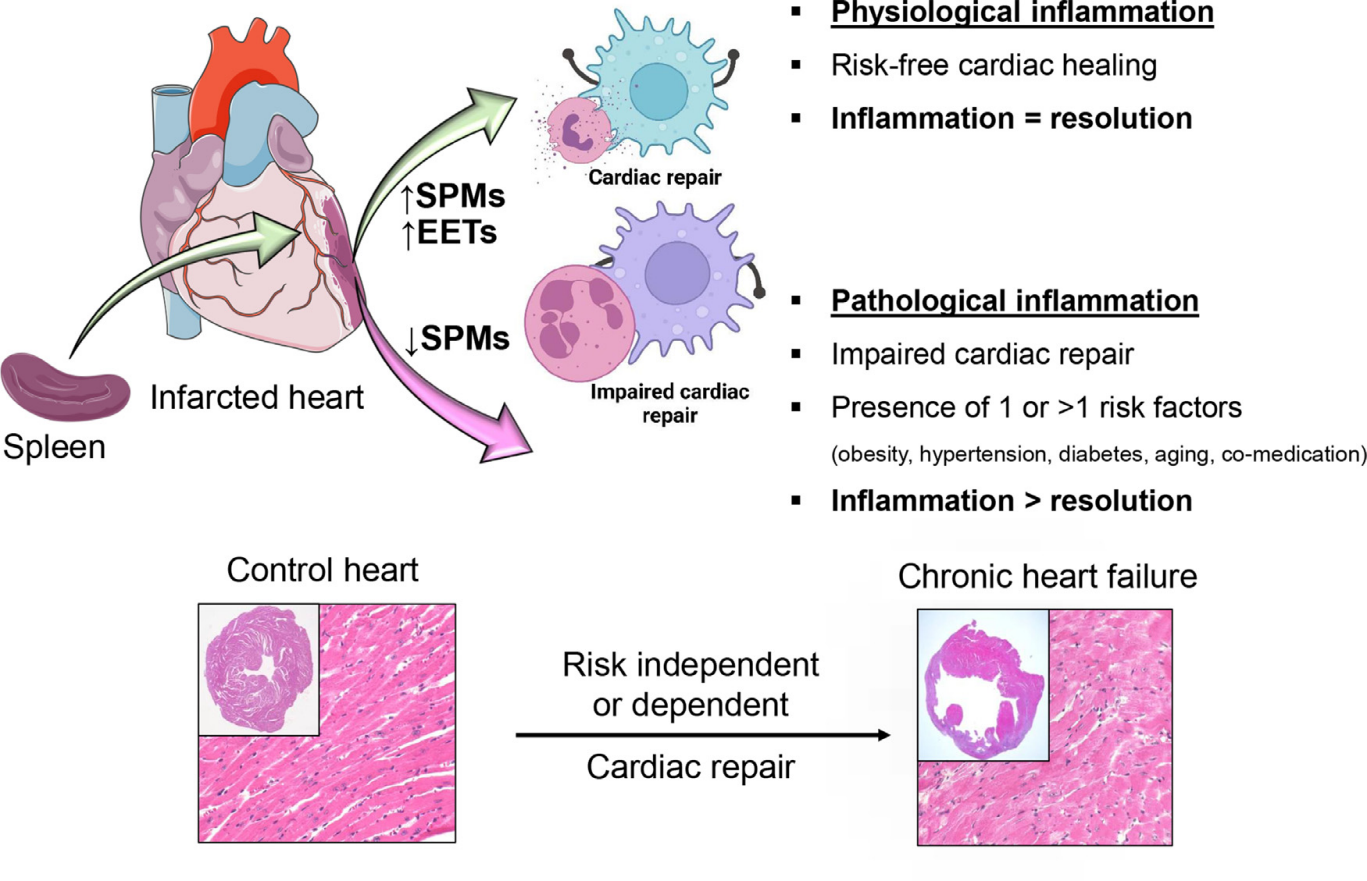
**SPMs direct cardiac repair and resolution of inflammation after cardiac injury.** After ischemic cardiac insult to risk-free and healthy mice, splenic leukocytes orchestrate the biosynthesis of SPMs and facilitate cardiac repair. However, lifestyle-related diseases such as obesity, diabetes, and hypertension, dysregulate acute ‘physiological’ inflammation. After a cardiac injury, if acute inflammation remains unresolved, that advances to chronic ‘pathological’ inflammation leading to progressive heart failure. SPMs entity coordinate resolution process leading to cardiac repair.

The pathogenesis of cardiovascular disease includes both chronic, low-grade inflammation and acute inflammation (occurring in setting of acute coronary syndrome). The initial low-grade residual and chronic inflammation ensues as a response to failed inflammation clearance process that is common in individuals with the risk factor of metabolic defects including obesity, diabetes, and hypertension.^6^ In the acute coronary syndrome and myocardial infarction (MI), acute inflammation due to plaque rupture initiates subsequent response that may lead to more long-term cardiac damage. After the reperfusion with coronary intervention with stent or thrombolysis, there is variation in the degree of recovery of inflammation which has prognostic value in the development of ischemic HF. In the setting of prolonged severe inflammatory response, adverse remodeling occurs leading to HF. Majority of research thus far has been focused on direct inhibition of inflammatory pathways such as TNF-alpha antagonist or IL-1ß antagonist.^7^^,^^8^ However, the results have been modest. In contrast, a distinctive pathway called resolution pathway plays an important role in the recovery of inflammation. The resolution pathway is an active process that begins in parallel with inflammation. Resolution pathway can be explained as “eradication of *the products of inflammation”* or as a *“Cessation of inflammation without suppuration”.*^9^^,^^10^ The pathway of resolution is an active process mediated by production of pro-resolving lipid mediators (SPMs) and phenotypic changes of inflammatory cells into immune-quiescent phenotype.^11^, ^12^, ^13^ As such, the aim of this review is to identify inflammation and resolution- related inflammatory cells, signaling molecules, and mediators in the setting of acute coronary syndrome and subsequent HF (Figure 2).

## Inflammation initiation and resolution signaling in cardiovascular disease

One of the essential goals for treatment in setting of ischemic heart disease after acute coronary syndrome is designing an appropriate strategy to minimize myocardial necrosis, improve myocardium viability, and optimize cardiac repair to prevent HF. After the onset of MI, as the first line of defense, the splenic leukocytes mobilize to the injured heart for myocardial damage clearance, which is commonly perceived as an inflammatory response.^14^ This type of injury stimulation response is also associated with activation of inflammatory cell and endothelial cells, as well as reactive oxygen species (ROS), eicosanoids, and cytokine/ chemokine production after cardiac injury.^15^ Due to aging, the process of initiation of physiological inflammation impaired with marked dysregulation of resolution signaling molecules in an inter-organ pathways.^16^^,^^17^

### Activation of immune cells

Neutrophils are the first responders with a defensive role against inflammation for injury, infection, and stress-induced cellular dysfunction.^18^ However, during the sequential events of inflammation and resolution, neutrophils show differing phenotype and function. For example, neutrophils massively infiltrate in the myocardial tissue from the spleen after myocardial infarction, where they can promote both tissue repair and collateral damage processes. After a cardiac injury, neutrophils follow a temporal polarization pattern, switching from a pro-inflammatory N1 to an anti-inflammatory N2 profile.^19^ Neutrophils with N1 phenotype are dominant during the initial stages of MI, whereas N2 phenotype is observed during the resolution of inflammation and tissue repair.

Monocytes, generated from bone marrow, are stored in the spleen and eventually circulate under homeostatic conditions for 1–3 days.^20^ They differentiate into macrophages that reside in tissues. Macrophages have important role in inflammation and resolution during heart injury. Macrophage phenotypic plasticity and polarization direct the wound healing, fibroblast to myofibroblast differentiation, endothelial cells activation, removal of debris and tissue repair through releasing inflammatory chemokines and cytokines and recruitment of monocyte and neutrophil.^20^ In simplified version, macrophages have the following major classification- pro-inflammatory (M1) and pro-resolving or reparative (M2) phenotype. A recent study shows the important role of regulatory T cells (Tregs) to promote pro-resolving functions in atherosclerosis regression through suppression of ongoing macrophage and T-cell pro-inflammatory responses and re-education of macrophages to a pro-resolving state that facilitates tissue repair and plaque contraction. Lymphocytes from chronic HF patients suggest compromised cell immune modulation of CD8^+^ and CD4^+^ T cells suggestive of the defective pro-resolution pathway.^21^

### Release of cytokine and chemokines

Cardiac repair involves activation of several cytokines and adhesion molecules such as cytokines (TNF-α and Interleukin-1, -6). As one of the major inflammatory cytokines, TNF- α is shown to have bidirectional effects based on their receptors. For instance, one early study shows TNF- α contributes to development and worse prognosis of LV dysfunction, another shows that TNF- α actually impedes the apoptosis of cardiomyocytes inducing cytoprotective signals.^22^ Now it has been hypothesized that TNF-α exerts different functional effect via their two receptors - TNFR1 and TNFR2.^23^ All these findings suggest the complexity of the biological system in the repair process and highlight the role of cytokines in inducing inflammatory responses after MI. Another member of the cytokine family, ILs, can release numerous mediators such as growth factors, chemokines, cytokines, and adhesion molecules.^24^ IL-6 has both pro-inflammatory and anti-inflammatory properties and actively participates in hematopoiesis, acute phase, and immune responses.^24^ IL-6 activates target genes involved in differentiation, survival, apoptosis, and proliferation as well as protection of myocytes from apoptosis and promotion of LV hypertrophy.^5^ The close interaction between various immunological components to initiate inflammation in cardiovascular disease leads to worse outcome. In addition, the direct inhibition of inflammation is insufficient for optimal recovery and homeostasis from the initial insult.^21^ CCL-2 is a chemokine with role in HF by leading to dysfunction, fibrosis and adverse remodeling.^25^ However, it has role in both recruitment of pro-inflammatory macrophages but also regulatory T cell that has role in resolution of inflammation. In fact, 15-epi- LXA4 (15 epimer lipoxin A4) initiates resolution pathway after MI via rapid neutrophil clearance.^13^ Along with cytokines variety of matrix metalloproteinases (MMPs) participate in cardiac remodeling post-MI, out of many MMPs especially MMP-9 is the widely-studied MMP in HF after myocardial infarction.^26^^,^^27^ In the presented review, we focused on the distinct pathway for resolution of inflammation with emphasis on the biosynthesis of pro-resolving lipid mediators and their role in cardiac repair.

## Resolution of inflammation

Until recently, resolution of inflammation was thought to be a passive process, mediated by blocking and inhibiting inflammatory mediators, or dilution of inflammatory milieu, enzymes, and pathways.^9^ However, recent evidence points toward a distinctive pathway of resolution, where the bioactive lipid mediators (such as specialized pro-resolving mediators- SPMs) play an important role.^28^ Several key regulatory mechanisms have been described that mediate resolution, including pro-resolving lipid mediators (lipoxins, resolvins, maresins, protectins etc.) and phenotypic changes of inflammatory cells (neutrophils, macrophages, or T cells) into immune-quiescent phenotypel.^11^, ^12^, ^13^ More specifically, SPMs reduce neutrophil infiltration, decrease pro-inflammatory cytokines (TNF-α, Il-6) production by T cells, activate reparative cytokines (IL-10), and also stimulate phagocytic activity of macrophage. Therefore, targeting to enhance these resolution pathways may allow primary, secondary prevention of atherosclerosis, acute coronary syndrome and ischemic cardiomyopathy/HF.

### Apoptosis of neutrophils

The crucial factor for initiating resolution after MI damage is the clearance of apoptotic neutrophils from the injury site by macrophages through efferocytosis.^29^ For instance, pro-resolution annexin A1 (AnxA1) and lactoferrin released by apoptotic neutrophils limit their recruitment to the site of injury.^30^ We have previously shown that another pro-resolution mediator resolvin D1 (RvD1) leads to promote whole-cell phagocytosis of necrotic cells and "amplify eat me signal" of macrophages in the atherosclerosis.^28^ The macrophage that ingests the apoptotic neutrophils lead into immune-quiescent phenotype of macrophages and subsequent downstream effects. With the absence of appropriate clearance of apoptotic cells, it could lead to secondary necrosis and subsequent chronic inflammation.^31^

### Tissue repair and homeostasis

After an inflammatory injury, tissue repair and re-establishment of tissue functionality are crucial to return the system to homeostasis. Activated immune-quiescent alternative macrophages secrete anti-inflammatory and reparative mediators, including transforming growth factor β (TGF-β) and vascular endothelial growth factor (VEGF).^29^ TGF-β takes part in the repair and regeneration of tissues by inducing myofibroblasts generation and stimulation of regulatory T cells. These growth factors also boost the regeneration of cells and the synthesis of reparative proteins, facilitating tissue homeostasis without the formation of fibrosis or scar.^29^ Pro-resolution lipid mediators such as epoxyeicosatrienoic acids (EETs) are implicated in increased recruitment of dendritic cells and reparative monocytes leading to improved structural remodeling after ischemic insult.^32^

### Pro-resolving lipid mediators cascade

There are two types of fatty acids that produce immune responsive lipid mediators: conditional ω-3 (eicosapentaenoic acid- EPA and docosahexaenoic acid- DHA) and essential ω-6 (arachidonic acid -AA) fatty acids.^31^^,^^33^ The ω-3 and ω -6 metabolites are biosynthesized via various enzymes such as cyclooxygenases (COX), lipoxygenases (LOX), and cytochrome P450 monooxygenases (CYP). AA can metabolize into prostaglandin (PG) and leukotrienes (LTs) critical for initiation of acute inflammation which are pro-inflammatory but could also process into lipoxins (LX) which are pro-resolution mediators.^13^^,^^34^ In response to injury or infection, eicosapentaenoic acid (EPA) and docosahexaenoic acid (DHA) are mostly anti-inflammatory by producing resolvins, protectins, maresins and resolvins in time dependent manner during the acute inflammation provided COX/LOX/CYP are functional. These different lipid mediators play activate specific G-protein coupled receptor mediating pro-resolution pathway in different immune or non-immune cells.

## LOX, COX, and CYP-directed lipid mediators in inflammation and resolution

The various inflammatory cells mediate inflammation and resolution of injury through the lipid mediators and receptors. Arachidonic acid (AA) is one of the predominant precursors that generate both pro-inflammatory and anti-inflammatory mediators after the enzymatic pathway. AA (ω-6) is converted to leukotrienes (LTs) and lipoxins (LXs) via LOX pathway; converted to prostaglandins (PGs) and thromboxanes (TXs) via the COX pathway; and converted to the epoxyeicosatrienoic acids (EETs), dihydroxyeicosatrienoic acids (DHETs), and hydroxyeicosatetraenoic acids (HETEs) via the CYP pathway.^35^ The other type of fatty acid ω-3 is a precursors of EPA and DHA which produces pro-resolution mediators including resolvins and maresins via LOX pathway.^31^ LAMPs are lifestyle-associated molecular patterns that dysregulate immune response and lead to unresolved inflammation without resolution of inflammation to resolution, leading to chronic inflammation and immune cell-cardiac damage and related morbidity. AA-derived amplified signals of PGs, TXs, 12-HETE, and cholesterol crystals are examples of LAMPs, furthermore pro-inflammatory and resolution mediators are responsive to lifestyle factors such as diet, sleep, physical activity, smoking/vaping, and alcohol intake (Figure 1) that warrants additional investigation for cardiovascular health.^2^^,^^3^^,^^36^

### LOX pathway

LOXs regulate the hydroxylation of poly-unsaturated fatty acids (PUFA) and synthesis of bioactive lipid mediators both pro- and anti-inflammatory in cardiac injury.^37^ It has multiple subtypes, including 12-/15-LOX, 5-LOX. These enzymes exerts on different fatty acids to produce various mediators with different roles. AA-derived lipoxins A_4_ and B_4_ via 12- and 15-LOX enzymes balance the inflammatory effects such as neutrophil chemotaxis, promote resolution.^37^ Both the lipoxins counter-regulate the pro-inflammatory effects of leukotriene B_4_ and C_4_ that help lower neutrophil adherence to endothelial cells, vasoconstriction, and neutrophil-mediated vascular injury in mice. The lipoxins also facilitate the removal of dead/ apoptotic neutrophils through efferocytosis by macrophages. The absence of 12/15LOX in mice leads to effective resolution and cardiac healing, explained by a compensatory increase of anti-inflammatory EETs via CYP pathway that aids in cardiac repair.^35^ Particuarly, female mice shows improved HF survival characterized by differences in cardiac functional recovery and structure, more reparative immune cells and higher levels of EETs, signaling molecules with anti-inflammatory effects.^32^ DHA-derived resolvins are formed via a combination of 5-LOX and 15-LOX enzymes.^37^ Resolvins are classified into D- and E- series resolvins.^38^ RvD1 attenuates inflammatory responses by increasing resolving leukocytes, reducing pro-inflammatory cytokine secretion, control neutrophil swarming, and migration of quiescent monocytes leading to improved outcomes in myocardial infarction and also acts as a renoprotective mediator in HF.^12^^,^^39^, ^40^, ^41^ Maresin is formed from DHA via 12-LOX pathway.^42^ Maresin has multiple actions including reduction in neutrophil accumulation in response to injury, stimulation of phagocytosis and efferocytosis of apoptotic cells, acceleration of tissue regeneration at the site of injury as well as transitions macrophage phenotype to anti-inflammatory/ pro-resolving type. Thus, maresin is a potent mediator in regulating both local inflammatory responses and stem cell functions.

### COX pathway

**Figure 3:**
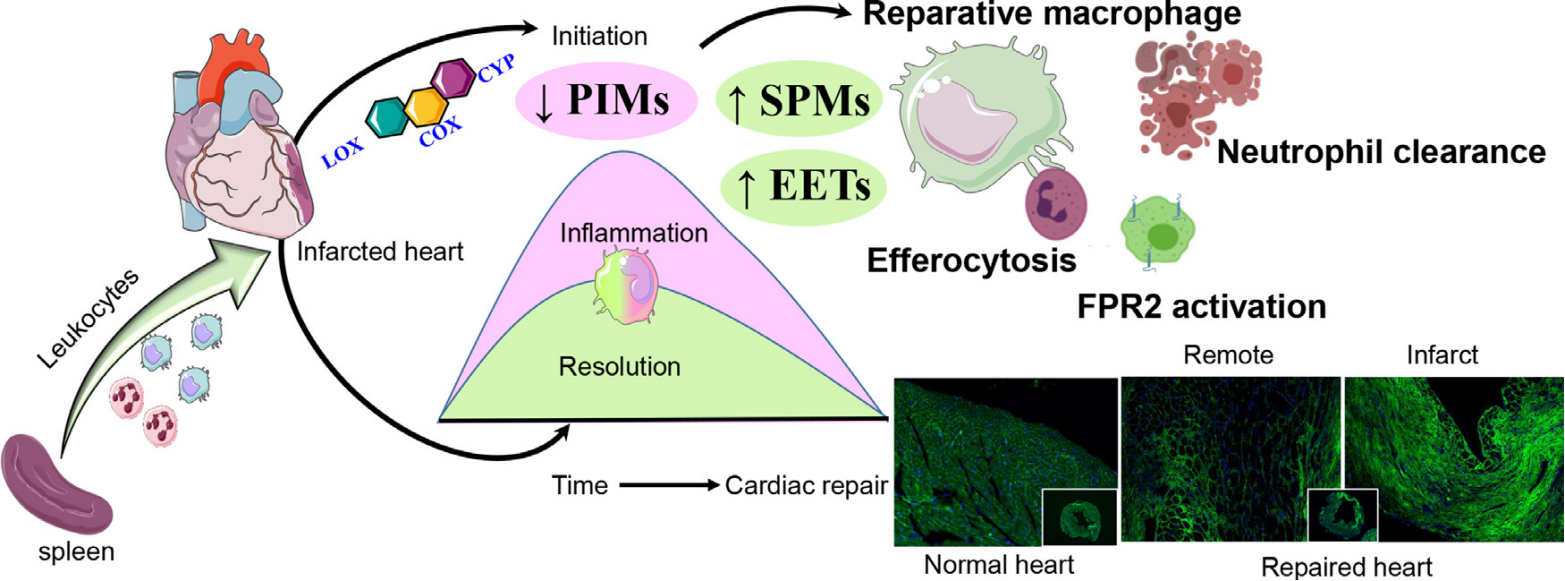
**Molecular and cellular signaling of resolution of inflammation in cardiac repair and heart failure.** In response to cardiac injury, splenic leukocytes (monocyte/macrophages) mobilize to the infarcted area, activate lipoxygenase, cyclooxygenase, cytochrome P450 enzymatic assembly. Leukocyte responsive SPMs and epoxyeicosatrienoic acids (EETs) are biosynthesized to facilitate the cardiac repair and safe clearance of inflammation (resolution) marked with macrophage-directed on-time efferocytosis and safe clearance of neutrophils. Dysregulation or imbalance of either pro-inflammatory mediators (PIM) or SPMs or EETs leads to impaired cardiac repair, which is common in obesity and or aging or in the absence of resolution receptor (FPR2) that is necessary for SPMs cardiac repair action.

Cyclooxygenase or COX occurs in two isoforms, constitutive isoform (COX-1) and the inducible isoform (COX-2). COX converts AA to prostaglandins (PGs) and thromboxanes (TXs). COX-1 synthesizes prostaglandins (PGs) from AA at low levels and maintains physiological functions. In comparison, COX-2 responds to sterile injury or pathogenic stimuli, cytokines, and mitogens.^43^ These metabolites take part in the initiation of inflammation, stimulate leukocyte infiltration, and over amplified in infarcted heart.^44^ We have previously shown that carprofen a non-steroidal anti-inflammatory drug (NSAID) hindered the resolution process in cardiac repair due to impaired macrophage phagocytosis and leukocyte clearance (Figure 3).

### CYP pathway

The catalytic reaction of cytochrome P450 epoxygenases to the AA leads to the synthesis of the eicosanoids such as epoxyeicosatrienoic acids (EETs), dihydroxyeicosatrienoic acids (DHETs), and hydroxyeicosatetraenoic acids (HETEs). In ischemia-reperfusion injury, EETs upregulates blood flow through the coronary and protect the myocardium from injury. In fact, we have shown that 12/15 LOX null mice fed with PUFA after permanent coronary artery ligation had improved signs of cardiac healing through higher leukocyte clearance with ultimately improved survival compared to wild-type mice.^45^ This outcome suggests that pro-resolving EETs were derived from PUFAs through the CYP450 pathway in the absence of the 12/15 LOX enzyme. Thus, cytochrome P450 epoxygenase-derived endogenous mediators may be a effective therapeutic agents for treating vascular inflammatory disorders. The different types of specialized pro-resolving molecules are summarized in Table 1.

**Table 1:** List of preclinical and clinical studies indicating SPMs and eicosanoids roles in cardiovascular medicine.

SPMs action in preclinical studies of ischemic heart disease			
Year	Specific SPMs in Disease Pathology	Mechanism of Action of SPMs	Refs.
2017, 2018	LXA_4_/ AT-LXA_4_ (Myocardial Infarction)	↑ activation of FPR2 *receptor* ↓ left ventricular remodeling ↑ mobilization of cytosolic calcium ↓ activation of the calcium-sensitive kinase ↑ calcium/calmodulin-dependent protein kinase II	^13^^,^^14^
2015, 2017, 2020	RvD1 (Myocardial Infarction and Muscle Injury)	↑ activation FPR2 *receptor* ↓ neutrophil swarming ↑ macrophage clearance ↓ muscle inflammatory cytokines ↓ pro-inflammatory macrophage infiltration ↓ LV remote hypertrophy	^12^^,^^39^^,^^58^
2019, 2020	Maresins (Myocardial Infarction)	↑ macrophage phagocytosis and efferocytosis ↓ infarct size and inflammatory mediators	^32^^,^^56^
2014	18 HEPE (Pressure overload-induced inflammation and fibrosis)	↑ generation of cardiac fibroblasts ↑ macrophages clearance	^59^

SPMs roles in Human Studies			
Year	Disease Pathology	Mechanism of Action of SPMs	Refs.
2019	Acute Myocardial Infarction	↑ physiological initial inflammation and ↑ SPMs activation prior to troponin during the onset of MI confirmed by ST elevation	^49^
2020	Acute Myocardial Infarction	↑ RvE1 in white patients than black patients after MI ↑ activation of resolution and inflammation after MI and marked by SPMs biosynthesis	^50^
2019, 2020	Peripheral Artery Disease	↑ pro‐resolution phenotype of leukocytes ↑ resolving effects through biosynthesis of SPMs	^60^
2020	Hypertension/Borderline CVD	↑ eicosanoids derived from arachidonic acids are pro-inflammatory (12 HHTrE, TXA2, TXB2 etc.) ↑ ω‐3 metabolites are crucial for the regulation of blood pressure and inflammation	^61^

**Abbreviations:** RvD1; Resolvin D1, LXA4/ AT-LXA4; lipoxin A_4_ and aspirin-tirggered lipoxin A_4_, FPR2; Formyl-peptide receptor-2, 18 HEPE; 18-hydroxyeicosapentaenoic acid, TXA_2_; Thromboxane A2, TXB2; Thromboxane B2.

## Leukocyte dynamics and macrophage directed resolution pathway

### Neutrophils dual phenotype and function

After the acute insult, the neutrophils release various “find me” signals to attract monocytes. Then, the neutrophils express “eat me” signals leading to the clearance of apoptotic neutrophils. In addition, apoptotic cardiomyocytes are opsonized through mesenchymal cell derived exosomes. Particularly, MFGE8 enhances opsonization of myocardial debris and also activates phagocytic signaling that facilitate removal of apoptotic cells after ischemic injury.^46^ Apoptotic neutrophils express different receptors (GPCR or G-protein-coupled ALX/FPR2), which are required for the functionality of pro-resolving mediators during the resolution process, such as lipoxins, resolvins, protectins.^18^ SPM entities, such as resolvin D1, and aspirin-triggered lipoxins, promote clearance of leukocytes from the infarcted site, thus promote the resolution of inflammation and cardiac repair this in turn changes the phenotype of macrophages from pro-inflammatory to anti-inflammatory and initiates the pathway of resolution.

### Role of macrophages in resolution of inflammation

During the first 24 h after MI, almost half of the monocytes are infiltrated to the injury site and differentiate into pro-inflammatory M1 macrophages and take part in efferocytosis.^47^ During the next 3–7 days, the infarcted myocytes, neutrophils forming a necrotic zone upregulates the pro-resolving macrophages which promote resolution of inflammation via cardiac remodeling and healing. This is in part mediated by synthesis of SPMs that orchestrate the resolution of inflammation at the injury site and in the spleen. Therefore, the entire inflammation-resolution signaling pathway is highly complex, time-sensitive, LOXs activation, and substrate dependent, and involves several factors starting from (i). timely leukocyte activation, and participating in cell apoptosis, (ii). clearance of dead cells by macrophages, (iii). biosynthesis of specialized lipid mediators and phenotypic changes of macrophages from pro to anti-inflammatory for effective efferocytosis and (iv). activation of the receptors for the interaction with specific resolving lipid mediators to accomplish the resolution process (Figure 3). All these cumulatively coordinate the cardiac healing and repair after myocardial injury.

## Other cardiovascular disease and pathway of resolution

In addition to the ischemic heart disease including myocardial infarction and HF, there are other cardiovascular conditions associated with chronic inflammation in which resolution pathway has been elucidated including heart failure with preserved ejection fraction. Aortic aneurysm is a disease characterized by aortic dilatation, with potential for rupture which may cause rupture which is often fatal. It is due to chronic inflammation related to chronic recruitment and activation of neutrophil. A stop signal mediated by lipoxin formation from 12/15 lipoxygenase has shown to prevent progression of aortic aneurysm.^48^

## Clinical and therapeutic implication of inflammation and resolution pathway in ischemic heart disease

The pathway of inflammation and resolution in ischemic heart disease in human is undergoing active investigation. We and others have shown that myocardial infarction causes temporal changes in the level of specialized pro-resolving molecules.^49^ It also causes differential levels of resolution mediators including resolvin E1 based on sex and ethnicity.^50^ However, biomarkers related to pathway of resolution on long-term clinical outcomes after MI or CHF is currently lacking.

Therapeutically, efforts to directly inhibit pathways of inflammation confers neutral or even worse outcomes in various clinical trials. For example, TNF-alpha inhibitor etanercept failed to show mortality benefit in HF patients.^51^ The CANTOS (Canakinumab Antiinflammatory Thrombosis Outcome Trial) trial investigating canakinumab (IL-1ß monoclonal antibody) showed reduced recurrence of cardiovascular event after myocardial infarction, but was associated with increased infection.^7^ The COLCOT (Colchicine Cardiovascular Outcomes Trial) trial investigated the use of low-dose colchicine led to decrease in composite primary outcome, but individual components of the primary outcome, including recurrent myocardial infarction or cardiovascular death, was not statistically significant.^52^

There is on-going debate on the benefit of ω-6 and ω-3 fatty acid in cardiovascular disease. The recent REDUCE-IT (Reduction of Cardiovascular Events with Icosapent Ethyl–Intervention Trial) trial showed improved outcome with icosapent ethyl in setting of primary prevention of CAD.^53^ The improved outcomes may be due to different ratio and absolute content of subtype of ω -3. More specifically, the high ratio of EPA to docosahexaenoic acid (DHA) in REDUCE-IT trial may contribute to the benefit seen. However, the study's placebo was mineral oil, which has worse cardiovascular outcome, rather than “neutral placebo” effect. The pro-resolving properties of EPA and DHA-derived (from ω-3 fatty acids) lipid metabolites open up a new avenue in the field of novel therapeutics and serve as immunoresolvents and pro-resolving, not immunosuppressive.^54^ Most strikingly, SPMs are potent specific GPCR agonists, and approximately 40% of all approved drugs activate this receptor class. Similar to past drug development successes for pro-inflammatory agents, it can be suggested that future works on pro-resolving mediators also need a similar approach that primarily includes understanding their biological roles in basic biomedical research. In this context, the new quantitative findings based on chromatography-targeted lipidomics will continue to flourish and provide novel approaches for preventing CVDs and treating HF patients. Recent discoveries shed light on how aspirin and aspirin-like drugs work and why they control inflammatory pain.^55^ For many years, inflammation was thought to be resolved after removing inflammatory mediators leading to the re-establishment of tissue function. More than 20 years after Ferreira and Vane's discoveries, it was recognized that endogenous SPMs are produced to block neutrophil recruitment and resolve inflammation.^38^ Thus, resolution of inflammation is an active and time-dependent mechanism with a biosynthetic shift from pro-inflammatory to pro-resolution mediators, the SPMs. Based on these emerging roles of SPMs and cys-SPMs, the bioactive mediators are uniquely positioned to have a role in many human diseases associated with inflammation because of their primary actions on effector cells in the innate immune system, including both neutrophils and macrophages.^56^^,^^57^

## Conclusions

Physiological acute inflammation is essential for cardiac repair and defense mechanisms after injury or infection. However, if resolution of inflammation is suboptimal that leads to chronic inflammation and subsequent advanced HF. Cardiometabolic defects such as obesity, hypertension, and diabetes are underlying causes for the genesis of sustained and suboptimal inflammation. Given the complex interaction of essential fatty acids, enzymes, lipid mediators, along with their role in pathophysiology of ischemic heart disease and HF, there will be continued effort in finding therapeutic targets which may allow development of novel personalized and precise pro-resolution therapeutics application. Further an integrative research is warranted on LOXs activation, and inactivation, substrate requirement, and SPMs receptor specific action for personalize and precise treatment in cardiac repair and HF.

## Outstanding questions

Resolution of inflammation is now recognized as an active process rather than passive event necessary for tissue repair and safe clearance of deceased cell debris after injury like ischemic event (heart attack). Unfortunately, markers that differentiate inflammation (eicosanoids) and resolution (SPMs) are biosynthesized simultaneously and need challenging state-of-the art technological procedures/methodology. Treatment feasibility using resolution mediators is established in rodent models and few clinical studies determined the inflammation-resolution signaling in patient samples. There is still uncertainty, scope, and opportunity to treat patients using bioactive resolution molecules. Co-medication(s) and co-medication related suppression or activation of resolution molecules in clinical setting warrants additional investigation. Future, clinical studies focusing on resolution biomolecules quantitation specifically in setting of lifestyle dysregulation such as diet, sleep, and exercise with mechanism-based pre-clinical studies are necessary for successful advancement in inflammation and resolution physiology.

## Search strategy and selection criteria

Data for this review were collected using PubMed searches between 2000 and 2022, using the terms “*resolution of inflammation”, “cardiac repair”, “lipoxygenase and cardiac repair”, “resolution of inflammation and heart failure”, “unresolved inflammation”, “resolution of inflammation and myocardial infarction”, “resolution of inflammation and heart attack”, “macrophages and specialized pro-resolving mediators (SPMs) in cardiac repair”, “SPMs and heart failure.*

## Contributors

G.V.H.: conceptualization, revision, eediting, writing, approval, D.H.L: Literature search, writing.

## Declaration of interests

No conflict of interest.
